# Steroid-Dependent Nephrotic Syndrome With Remission After Rituximab Implementation in a 14-Year-Old Boy: A Case Report

**DOI:** 10.7759/cureus.91845

**Published:** 2025-09-08

**Authors:** Grazyna Waska, Katarzyna Pielorz-Janiczek, Andrzej Badeński, Anna Strozak, Magdalena A Miernik-Skrzypczak

**Affiliations:** 1 Internal Medicine, Specialist Hospital No. 1 in Bytom, Bytom, POL; 2 Pediatric Nephrology, Medical University of Silesia, University Clinical Hospital No. 1, Zabrze, POL; 3 Internal Medicine, Dr. Anna Gostynska Wolski Hospital, Warsaw, POL; 4 Pulmonology and Hematology, Lower Silesian Center of Oncology, Wrocław, POL

**Keywords:** fsgs, nephrotic syndrome, pediatrics, rituximab, steroid-dependent

## Abstract

Steroid-dependent nephrotic syndrome (SDNS) in children poses significant therapeutic challenges due to frequent relapses and the risks linked to prolonged immunosuppressive treatment. We present a case of a 14-year-old boy diagnosed with steroid-sensitive nephrotic syndrome onset at three years old who progressed to steroid dependence with multiple relapses despite the therapy with calcineurin inhibitors and mycophenolate mofetil. The patient developed complications including growth retardation, Cushingoid features, and persistent hypertension. Renal biopsy confirmed focal segmental glomerulosclerosis (FSGS), which is a histological variant associated with poor treatment response. After limited success with conventional therapies administered from November 2013 to December 2024, rituximab was initiated, resulting in sustained remission and enabling complete withdrawal of immunosuppressive agents. Post-rituximab, the patient also received growth hormone therapy, which contributed to improved growth and overall clinical condition. This case highlights the complexity of managing SDNS with FSGS and supports rituximab’s role in refractory cases, while underscoring the importance of monitoring for adverse effects.

## Introduction

Idiopathic nephrotic syndrome (INS) is one of the most common glomerular disorders in children, characterized by proteinuria, hypoalbuminemia, and edema caused by altered permeability of the glomerular filtration barrier to plasma proteins, leading to their loss, with an unknown etiology. Corticosteroids are the first-line treatment, and most patients respond with remission following initial therapy [1]. According to the recommendations of the Polish Society of Pediatric Nephrology (PTNFD), prednisone at 60 mg/m²/day (or 2 mg/kg/day) for four weeks is the first-line treatment, with extension to six weeks if remission is not achieved, followed by a tapering regimen over the next eight weeks [2]. Similarly, Kidney Disease: Improving Global Outcomes (KDIGO) guidelines recommend oral glucocorticoids given daily for four to six weeks followed by alternate-day dosing for an equal duration, highlighting standardized dosing regimens for initial therapy [3]. However, relapses are common, and up to 50% of cases progress to frequent relapses or steroid dependence, requiring prolonged immunosuppressive therapy to maintain remission [4].

Steroid-dependent nephrotic syndrome (SDNS) is defined by experiencing two or more consecutive relapses while on corticosteroid therapy or within 14 days after its discontinuation. This condition affects approximately 40%-50% of pediatric patients. Despite the availability of several second-line treatments, including levamisole, mycophenolate mofetil (MMF, an immunosuppressive drug commonly used in nephrotic syndrome), and calcineurin inhibitors (CNIs, immunosuppressive agents such as cyclosporine or tacrolimus), management remains difficult due to the risk of steroid-related adverse effects such as growth retardation, Cushingoid features, and nephrotoxicity [5]. Both the underlying disease and its treatment contribute to long-term morbidity.

Focal segmental glomerulosclerosis (FSGS), a common histopathological variant of INS, refers to scarring within the kidney and is frequently associated with steroid resistance and poor renal outcomes. It is classified among podocytopathies - diseases primarily affecting podocytes, the specialized cells forming the glomerular filtration barrier. Although its pathogenesis is not fully understood, immune dysregulation is considered a key factor [6]. Rituximab, a monoclonal antibody targeting CD20, has shown promise in treating complicated INS, particularly in patients with frequent relapses or steroid dependence. While long-term remission rates vary and relapses are common, rituximab has demonstrated efficacy in inducing sustained remission and reducing corticosteroid exposure [7].

Steroid resistance affects approximately 10%-15% of pediatric patients with nephrotic syndrome and is associated with a high risk of progression to end-stage kidney disease when unresponsive to alternative immunosuppressive treatments [8]. Reports suggest an increasing prevalence of steroid-resistant and histologically severe forms, such as FSGS, in some populations, highlighting the need for effective, tailored treatment approaches [9]. This study presents the case of a 14-year-old boy diagnosed with SDNS at three years of age, who experienced multiple relapses despite intensified therapy, developed secondary complications, and ultimately achieved remission following rituximab treatment. What makes this case distinctive is the unusually long disease duration prior to sustained remission, the recovery of growth following combined rituximab and growth hormone (GH) therapy, and the need to address psychiatric complications alongside nephrological management.

## Case presentation

In November 2013, a three-year-old boy was referred to the pediatric nephrology department from a regional hospital due to progressive peripheral edema and declining renal function during his first episode of nephrotic syndrome. On admission, the patient appeared acutely ill, with significant edema and reduced activity. Physical examination revealed symptoms of an upper respiratory tract infection, including cough and nasal congestion without fever. The patient had a history of fever one week prior to hospital admission. Additionally, decreased vesicular breath sounds were noted at the lung bases, along with a soft systolic murmur and massive generalized edema involving the eyelids, extremities, scrotum, and abdominal wall. The patient demonstrated a weight gain of 4.2 kg attributable to edema upon admission. Laboratory evaluation was consistent with new-onset nephrotic syndrome, showing hypoalbuminemia, nephrotic-range proteinuria, hypercholesterolemia, hypertriglyceridemia, hyperuricemia, anemia, and impaired renal function, with an estimated glomerular filtration rate (eGFR) of 29 mL/min/1.73 m². Coagulation studies revealed a prothrombotic tendency with prolonged activated partial thromboplastin time (APTT) of 79 seconds, prothrombin time at 85%, and elevated D-dimer levels of 2.44 mg/L (normal <0.5 mg/L). Ultrasonography demonstrated bilateral pleural effusions (approximately 2 cm), a small pericardial effusion, and minimal ascites. The patient was started on intravenous methylprednisolone (2 mg/kg/day), broad-spectrum antibiotics, anticoagulant prophylaxis, and supportive care including omeprazole, calcium carbonate, and vitamin D3. Due to evidence of prerenal acute kidney injury secondary to hypoalbuminemia, treatment included intravenous 20% albumin and diuretics (furosemide). Antihypertensive therapy was initiated for elevated blood pressure. Endocrinologic evaluation revealed secondary hypothyroidism, and levothyroxine therapy was commenced.

**Figure 1:**
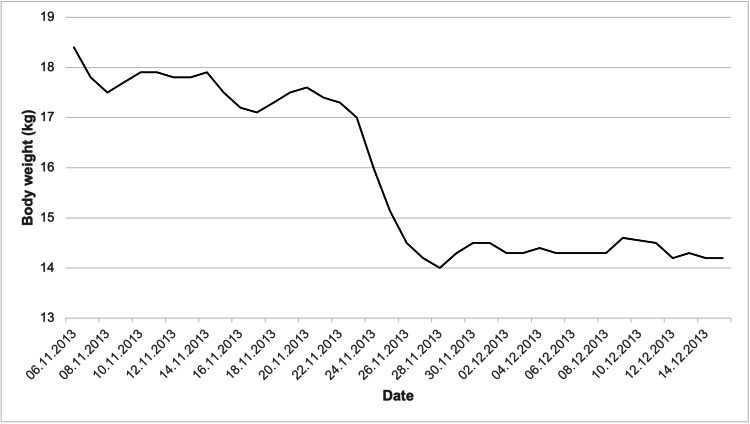
Changes in body weight during edema resolution

After five weeks of corticosteroid therapy, the patient achieved partial remission, with improved renal function and resolution of edema, accompanied by a marked reduction in body weight due to fluid loss (Figure 1). He was discharged under regular outpatient nephrology follow-up. Six months later, one week after discontinuation of corticosteroids, he developed an upper respiratory tract infection followed by a relapse of nephrotic syndrome, presenting again with generalized edema and abnormal laboratory findings. Intravenous corticosteroids and supportive measures were initiated. Due to delayed response, cyclosporine A was added on day five of hospitalization, leading to remission. The patient was discharged with continued outpatient care.

Between 2014 and 2016, after the initiation of cyclosporine A therapy in May 2014, the patient experienced multiple relapses, each associated with viral upper respiratory or gastrointestinal infections. All episodes required hospital admission and full-dose corticosteroid therapy to induce remission. In March 2017, he presented with acute kidney injury following concurrent administration of cyclosporine A and clarithromycin. Laboratory evaluation revealed elevated cyclosporine level and reduced eGFR (38 mL/min/1.73 m²). Both drugs were discontinued, and treatment included intravenous fluids, oral corticosteroids, and empiric intravenous antibiotics. After recovery of renal function, cyclosporine was reintroduced with close monitoring.

In early 2018, the patient experienced two additional relapses requiring high-dose corticosteroid therapy. In June 2018, he was hospitalized due to behavioral disturbances, including aggression and self-injurious behavior. Neurologic evaluation was unremarkable. Psychological assessment indicated an adjustment disorder, and chlorprothixene was initiated following psychiatric consultation. Chronic corticosteroid use was considered a contributing factor. Despite therapeutic cyclosporine levels, disease control remained suboptimal, and tapering of steroids was unsuccessful. Antihypertensive treatment was modified due to sustained hypertension.

In 2022, he continued to experience frequent infections and steroid-dependent relapses. Physical examination revealed significant growth retardation (<3rd percentile), Cushingoid appearance, gingival hypertrophy, and persistent hypertension. Endocrine evaluation was initiated. Renal biopsy was performed and revealed FSGS. Due to inadequate clinical response to CNIs, MMF was added in February 2023. Endocrinologic investigations showed delayed bone age, normal GH stimulation, and normal pituitary magnetic resonance imaging, excluding somatotropic axis deficiency.

In December 2023, the patient was admitted for initiation of recombinant human GH therapy due to persistent short stature. The treatment was funded privately. However, frequent relapses necessitating full-dose corticosteroids led to temporary discontinuation of GH therapy, given concerns regarding potential adverse metabolic effects from concurrent use of corticosteroids and CNIs. Despite triple immunosuppressive therapy (cyclosporine A, MMF, and corticosteroids), disease activity remained poorly controlled, and corticosteroid doses could not be tapered below 20 mg every 48 hours. Additional attempts with levamisole were unsuccessful.

**Figure 2:**
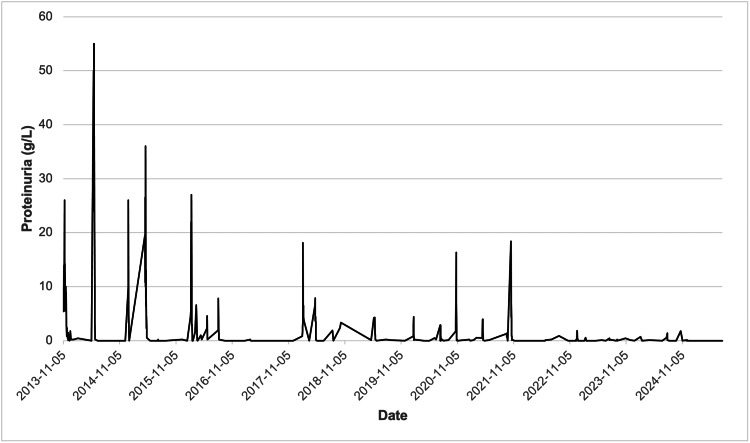
Changes in proteinuria during the course of treatment Peaks correspond to relapse episodes, while periods of near-zero proteinuria indicate remission.

**Figure 3:**
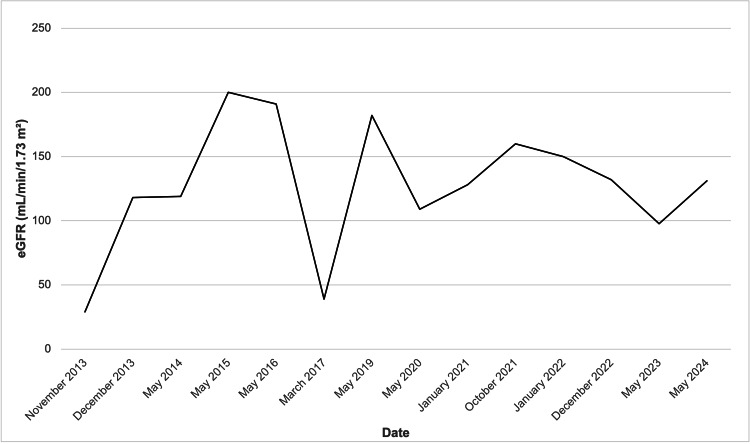
Changes in eGFR over time in the patient Early fluctuations reflect impaired renal function during relapses, with eventual stabilization after rituximab therapy. eGFR: estimated glomerular filtration rate

**Figure 4:**
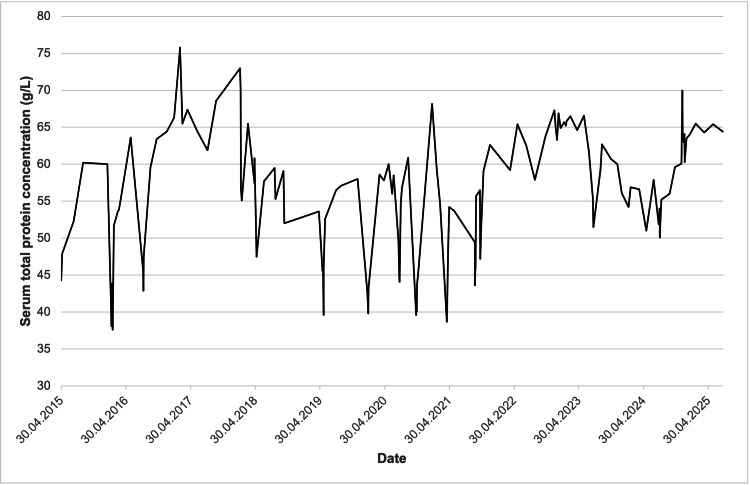
Changes in serum total protein concentration over the treatment period Values fluctuated in parallel with disease activity, stabilizing at higher levels after sustained remission post-rituximab.

In December 2024, rituximab therapy was initiated after obtaining informed parental consent, with two doses of 500 mg administered two weeks apart. The patient achieved sustained remission following administration, allowing complete withdrawal of corticosteroids and other immunosuppressive agents. Longitudinal monitoring of proteinuria, eGFR, and total serum protein levels over the 12-year follow-up period (Figures 2-4) illustrates the fluctuating course of the disease prior to rituximab and subsequent stabilization. Proteinuria exhibited intermittent peaks corresponding to relapse episodes, followed by periods of complete remission. eGFR was markedly reduced during early relapses (below 50 mL/min/1.73 m²) but improved significantly during remission phases, eventually stabilizing between 100 and 150 mL/min/1.73 m² from 2020 onwards. Total serum protein levels showed variable fluctuations during the disease course, reflecting both relapse activity and periods of recovery, but overall tended to stabilize in the range of 50-70 g/L after 2022. These trends highlight the clinical impact of rituximab in achieving sustained disease control and improved renal function. Follow-up is ongoing with a multidisciplinary team, including nephrology, endocrinology (continued GH therapy), psychiatry, and psychological services. Clinical condition and growth parameters have shown progressive improvement.

## Discussion

Our patient initially presented with the typical steroid-sensitive form of INS, which accounts for the majority of pediatric cases. However, following multiple relapses requiring repeated corticosteroid courses, he developed SDNS, a condition associated with increased morbidity and risk of long-term complications [6].

Prolonged glucocorticoid exposure resulted in classical manifestations of steroid toxicity, including growth retardation, Cushingoid features, weight gain, and arterial hypertension. These complications are frequently reported in children with SDNS undergoing chronic or recurrent steroid therapy and may significantly impair quality of life and development [5].

Despite sequential use of second-line immunosuppressive agents, including CNIs and MMF, disease control remained suboptimal. A renal biopsy was therefore performed, revealing FSGS - a histopathological finding associated with poor steroid responsiveness and higher risk of progression to chronic kidney disease [6]. Both PTNFD and KDIGO guidelines recommend renal biopsy in steroid-resistant cases, or in patients older than 12 years, as well as when other features such as nephritic syndrome or impaired renal function are present [2,3].

Following biopsy confirmation of FSGS and limited efficacy of multiple therapies, corticosteroid tapering was unsuccessful, with relapse upon dose reduction. Rituximab was subsequently initiated as a rescue therapy after failure of standard immunosuppressive strategies. Current international pediatric nephrology guidelines recommend rituximab in cases of steroid-dependent or steroid-resistant nephrotic syndrome unresponsive to conventional agents. Retrospective studies have shown that 30% to 80% of patients may achieve complete or partial remission following rituximab administration.

In the present case, rituximab therapy induced sustained remission and allowed discontinuation of corticosteroids and other immunosuppressants. This outcome aligns with growing evidence supporting rituximab as an effective option in complicated nephrotic syndrome, including in patients with biopsy-proven FSGS [7]. Compared with published pediatric FSGS cases treated with rituximab, the patient’s sustained remission appears notably long, highlighting the potential for rituximab to achieve durable disease control. Nevertheless, the safety profile of rituximab requires careful consideration in pediatric populations.

Adverse effects associated with rituximab include infusion reactions such as skin rash, fever, and dyspnea, infections, and hematologic abnormalities such as late-onset neutropenia. Although rare, severe complications like hepatitis B reactivation, *Pneumocystis jirovecii* pneumonia, and progressive multifocal leukoencephalopathy have been reported. Hypogammaglobulinemia, including persistent forms, may develop in a significant proportion of patients and can persist beyond one year, increasing the risk of severe infections such as agranulocytosis-related sepsis [8,10]. Children under the age of 10 may be more vulnerable to hematologic toxicity, warranting close monitoring during and after treatment [8].

This case illustrates the therapeutic challenges in managing pediatric SDNS complicated by FSGS. Rituximab may represent a valuable treatment option in refractory cases, facilitating sustained remission and reducing corticosteroid exposure. However, the risk of adverse events highlights the need for further long-term safety data and careful patient selection. Preventive strategies, including infection prophylaxis and immunoglobulin level monitoring, are essential during and after rituximab therapy [11].

## Conclusions

This case underscores the clinical challenges associated with managing SDNS with underlying FSGS in a pediatric patient. Rituximab was used following limited response to other immunosuppressive agents and was associated with sustained remission. Its role in difficult-to-treat cases is supported by current evidence, though careful monitoring remains essential due to the potential for adverse effects. Based on our experience, early consideration of rituximab in refractory SDNS may help prevent years of morbidity related to prolonged corticosteroid and CNI therapy. Importantly, successful disease control in this patient allowed withdrawal of long-term corticosteroid therapy, leading to gradual improvement in growth, weight reduction, and initiation of puberty. The outcome also highlights the value of a multidisciplinary approach, as coordinated care involving nephrology, endocrinology, and psychiatry contributed to long-term stabilization. Continued follow-up will be required to monitor the durability of remission and optimize developmental outcomes.
